# Current Evidence on Bisphenol A Exposure and the Molecular Mechanism Involved in Related Pathological Conditions

**DOI:** 10.3390/pharmaceutics15030908

**Published:** 2023-03-10

**Authors:** Ylenia Della Rocca, Enrico Matteo Traini, Francesca Diomede, Luigia Fonticoli, Oriana Trubiani, Alessia Paganelli, Jacopo Pizzicannella, Guya Diletta Marconi

**Affiliations:** 1 Department of Innovative Technologies in Medicine & Dentistry, University “G. d’Annunzio” Chieti-Pescara, Via dei Vestini 31, 66100 Chieti, Italy; 2 PhD Course in Clinical and Experimental Medicine, University of Modena and Reggio Emilia, Via del Pozzo 71, 41125 Modena, Italy; 3 Department of Engineering and Geology, University “G. d’ Annunzio” Chieti-Pescara, Viale Pindaro 42, 65127 Pescara, Italy

**Keywords:** mesenchymal stem cells, endocrine disruptors, bisphenol A

## Abstract

Bisphenol A (BPA) is one of the so-called endocrine disrupting chemicals (EDCs) and is thought to be involved in the pathogenesis of different morbid conditions: immune-mediated disorders, type-2 diabetes mellitus, cardiovascular diseases, and cancer. The purpose of this review is to analyze the mechanism of action of bisphenol A, with a special focus on mesenchymal stromal/stem cells (MSCs) and adipogenesis. Its uses will be assessed in various fields: dental, orthopedic, and industrial. The different pathological or physiological conditions altered by BPA and the related molecular pathways will be taken into consideration.

## 1. Introduction

**Figure 1:**
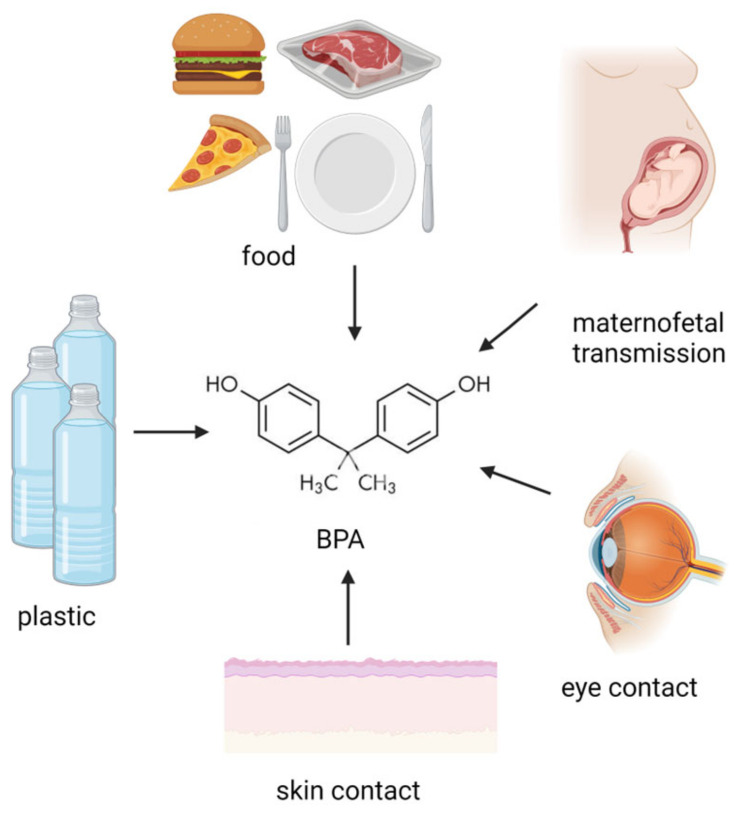
Potential BPA origin. BPA exposure sources involve ingestion, maternofetal transmission, inhalation, skin, and eye contact. (Created with BioRender.com).

**Figure 2:**
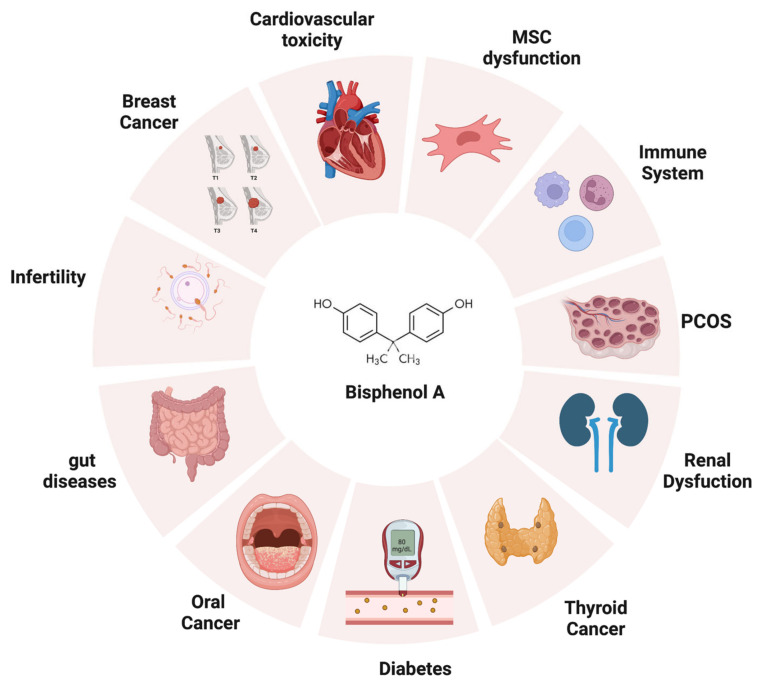
The adverse effects of bisphenol A on human health. BPA can negatively impact different targets once introduced into the human body, such as thyroid, heart, reproductive apparatus, gut, immune system, and kidneys (created with BioRender.com).

Endocrine disrupting chemicals (EDCs) are mostly exogenous artificial products characterized by the ability of interfering with the activity of the endocrine system. They have been described to be present as possible components of various materials (such as pesticides) as well as contaminants in food. The exposure to EDCs can occur by ingestion, inhalation, and skin absorption (see Figure 1). Based on the literature, EDCs do not only affect the endocrine system but also seem to increase the incidence of malignancy (e.g., breast cancer) as well as induce alterations in the immune response [1]. Moreover, EDCs have been found to be associated with reproductive function alterations, abnormal growth patterns, and neurodevelopmental delay [2]. EDCs seem to be transferred to the developing fetus and/or to the newborn through several different possible mechanisms, which can include trans-placentar transfer or breastfeeding (for EDCs secreted in breast milk). Children and pregnant women certainly represent particularly vulnerable populations to EDC exposure. Moreover, developmental exposure to EDCs may lead to the occurrence of possible side effects later in life, despite EDC-induced toxicity not being immediately evident after birth (see Figure 2). The action of endocrine disruptors is linked to their ability to totally or partially mimic the body’s natural hormones, such as estrogens, androgens, and thyroid hormones [3]. Their hormone-like action can lead to possible interferences between natural hormones and their receptors, therefore, altering hormonal axes. With regards to their action on hormone receptors, EDCs can trigger two types of responses: agonistic or antagonistic. Whenever EDCs exert an antagonistic action on hormone receptors in developing tissues and organs, developmental problems are one of the most worrisome complications, especially for the neurological and reproductive systems [4]. As for the exact mechanism of action leading to EDC-related comorbidities, recent studies reported a possible interference of these compounds with the apoptotic mechanisms in association to a direct effect on epigenetic regulation [5].

Among EDCs, Bisphenol A (BPA) is certainly of interest due to its environmentally significant concentration and the large variety of commonly used plastic-based materials containing it [6,7].

As with other EDCs, BPA directly interferes with several hormonal axes in the human body; therefore, giving reason of its potential pathogenic role in several diseases [8].

Of note, some of the most recent insights in BPA-mediated biological actions noted a central role for BPA-mediated impairment of adipogenesis in mesenchymal stromal/stem cells (MSCs). Most of the available literature aims at assessing the effects of one or more EDCs on a particular clinical setting, often focusing on one or few diseases [9]. The aim of the present review is to provide a comprehensive overview of the possible effects of EDCs on human health with a special focus on BPA-mediated action on MSCs.

## 2. Materials and Methods

A search was conducted in the PubMed/MedLine databases from inception to the present. The following search terms were used: BPA or bisphenol A, EDC or endocrine disrupting chemicals, MSC or mesenchymal stem cells, cancer, and health. All the major journals were indexed. Only journal articles were taken into consideration, while books and book chapters were excluded. Articles without full text electronically available and/or English translation were also excluded. After the removal of papers that did not focus on the aim of the present paper, we considered the articles referenced in the present review.

## 3. Main EDCs

### 3.1. Bisphenol A

Certainly one of the most studied EDCs is Bisphenol A (BPA), a synthetic organic compound characterized by two geminal phenol groups. BPA was initially developed as a synthetic estrogen in the late 19th century, but afterwards it was shown to be contained in plastics, polyvinyl chloride (PVC), epoxy resins, and dental sealants [10]. Currently, BPA is often found in polycarbonate plastics used for food packaging and bottles [11].

In 2002, the production of BPA was estimated to be 2.8 million metric tons and grew up to 5.5 million metric tons in 2011 [12].

Its increase can be attributed to its use as a monomer in the production of polycarbonate plastics, epoxy resins, and dental sealants [13].

BPA can be released either due to an incomplete polymerization or because of polymer hydrolyzation due to acid exposure, high temperature, or enzymatic digestion. Food and beverages are the main sources of BPA exposure, through accidental ingestion. Previous authors have already explored the possible interactions between BPA and its derivatives with the human body [14].

### 3.2. Other EDCs

#### Phthalates

Phthalates are esters of phthalic acid and represent a group of chemicals commonly found in plastics, where they are used with the aim of improving the flexibility and the strength of the material [15]. These compounds are also contained in many other products, such as detergents, adhesives, clothes, shampoos, hair sprays, and more [4]. The most common phthalates used are dimethyl-phthalate (DMP), di (2-ethylhexyl)-phthalate (DEHP), and dibutyl-phthalate (DBP). The accidental exposure to these compounds generally occurs through oral ingestion, by eating foods stored in plastic, or by breathing air polluted with phthalate particles. The exposure to these chemicals during pregnancy is notably associated with thyroid-hormone alterations and may also interfere [16] with sex steroid metabolism and production. Since sex hormones are fundamental for brain development, such interference is thought to be linked to autism spectrum disorders and/or neurological development delay [17].

### 3.3. Dioxins

Dioxins are a group of chemicals characterized by the presence of two benzene rings connected by a pair of oxygen atoms [18]. Different types of dioxins are distinguished based on the type of element bonded to the 8 carbon atoms on the rings, assigned numbers from 1 to 4 and from 6 to 9, respectively [19,20]. Dioxins are probably the most well-known type of EDCs and are a group of chemicals commonly contained in herbicides and pesticides. Dioxin toxicity can affect various organs and systems [21]. These chemicals, once assimilated by the organism, can last for a long time due to their stability and their affinity for lipid membranes, as well as their peculiar tropism for the fat tissue where they are stored. It is well documented that the dioxin half-life in the human body varies between 7 to 11 years [22]. The exposure of human subjects to high levels of dioxins in the short period may result in acute toxicity, with a sudden onset of skin manifestations (such as chloracne or skin hyperpigmentation), and impaired liver function. Based on the literature, the longer-term exposure to dioxins can cause endocrine dysregulation, immune system impairment, and nervous system damage [23]. The 2, 3, 7, 8-tetrachlorodibenzop-dioxin (TCDD), commonly referred to as simply “dioxin”, has been demonstrated to act as an endocrine disruptor [24]. Several studies highlighted impairment in ovarian steroidogenesis, inhibition of follicle development [25] and placental vascular remodeling [26], increased rates of spontaneous abortion [27], and reduced fertility linked to TCDD exposure [28].

## 4. Common Clinical Uses of Bisphenol A

### 4.1. Dentistry

In dentistry a lot of resin-matrix composites contain BPA derivatives in their matrix, including Bisphenol A-Diglycidyl Methacrylate (Bis-GMA), Ethoxylated Bisphenol A Glycol Methacrylate (Bis-EMA), Polycarbonate-modified Bis-GMA (PC Bis-GMA), and Bisphenol A Polyethoxy Methacrylate (Bis-MPEPP) [29,30]. It is well documented that the BPA-derivative weight percent in composites can range from 5 up to 20% for Bis-GMA, 1–5% for Bis-EMA, and varies between 5 and 10% for Bis-MPEPP [31]. BPA monomers are generally released from the composite matrix within the first 24 h, but such process may be prolonged due to further mechanical stimulation and/or chemical reactions [32,33]. Some studies, for example, found BPA release to be possibly connected to hydrolysis and catalysis by esterase in the human saliva [34]. Furthermore, the improper polymerization of the materials may enhance the release of BPA and its derivatives [35].

### 4.2. Orthopaedics

In the last years, the injection of polymeric cement has become a common practice in the orthopedic setting for the positioning of screw-in stabilizers in osteoporotic bones [36,37]. Screw-in stabilizers are also often required for patients with neuromuscular disorders to enhance pelvic stability. Bis–GMA composite resins, for example, have been used to improve the stability of the screws in the osteoporotic bone as reported by Dubory et al., 2015 [38]. The Bis-GMA has optimal mechanical properties in the absence of exothermic reactions during polymerization, which are instead typical of other screw augmentation techniques that use polymethylmethacrylate (PMM) [39]. However, some reports have been published on possible complications related to leakage from cemented vertebrae, such as pulmonary embolism [36,40].

### 4.3. Bisphenol in the Industrial Uses

BPA is the main raw material in the production of diglycidyl-ether BPA (DGEBA), an epoxy resin that is composed of BPA for more than 67% of its molar mass [41,42]. DGEBA is one of the most common types of epoxy resin, with excellent mechanical properties [43]. The use of the BPA in the manufacturing of DGEBA was reported to have detrimental effects on both human health and the environment [44]. Recent efforts to fully replace the BPA with other materials (such as lignin) are aimed at reducing BPA-related side effects [45].

## 5. Bisphenol A Effects on Human Health

### 5.1. Bisphenol A Effects on Immune System

BPA has been reported to interfere with some of the principal function of immune cells. Macrophages, for example, express two Estrogen Receptor (ER) isoforms, the ERα and ERβ. Due to its structural similarity to oestradiol, BPA can bind these receptors, therefore, activating hormone-mediated intracellular cascades in macrophages [46,47]. Moreover, other studies reported that BPA could stimulate the production of pro-inflammatory cytokines, such as IL-1, IL-6, and IL-12 [48]. BPA has also been reported to interact with antigen presenting cells. Dendritic cells (DCs), in particular, express Erα and Erβ [49]. DC exposure to BPA determines an increase in IL-5, IL-10, and IL-13 production [50], therefore, altering the immune response regulation and eventually inducing a switch towards Th2-type response. Granulocytes, the most common immune cells, can also be modulated by BPA [51]. Watanabe et al. evaluated the effect of BPA on the neutrophilic differentiation induced by dimethyl sulfoxide (DMSO) and granulocyte colony stimulating factor (G-CSF) [52]. The authors concluded that BPA may increase the production of the superoxide and neutrophil differentiation; however, they also observed that the treatment with tamoxifen, an estrogen receptor inhibitor, did not revert BPA-mediated effects, suggesting an ER-independent pathway [53].

Lymphocytes, the main actors of the adaptive arm of the immune system, express different hormone receptors that can regulate their differentiation [54]. Several studies stated that BPA can bind T cells affecting Th17, Th1, and Th2 differentiation [50]. BPA significantly increased the IFN-γ and IL-4 secretion in animal models, suggesting a possible upregulation of the Th1 response [55,56]. In a paper published by S. Yoshino et al. in 2005, mice treated with BPA prenatally were reported to have a Th1-cytokine secretion pattern [57]. BPA can also interfere with the production of antibodies, as demonstrated in murine models [58]; the same study also revealed that BPA increases the proliferation of splenocytes, spleen macrophages, and antibody production in a dose-dependent fashion. Lastly, previous studies demonstrated that gestational exposure to BPA can increase the production of specific IgG subtypes, confirming the attitude of the BPA to interfere with the immune system cells [59].

### 5.2. Bisphenol A and Type 2 Diabetes Mellitus

Both BPA and high estrogen levels may induce hyperinsulinemia and could be considered risk factors for type 2 diabetes mellitus (T2DM) [60,61]. In fact, BPA modulates alter insulin secretion by pancreatic islets through ER signaling on β-Cells [61]. Acute exposure to low doses of BPA in mice was found to determine a rapid decrease of glucose levels within 30 min from injection [62]. Alternatively, higher BPA doses were demonstrated to induce hyperinsulinemia in the absence of blood glucose level alterations. Based on these observations, BPA can be considered as an established inducer of insulin secretion [63]. However, exposure to BPA for eight days in murine models was described to inhibit basal insulin secretion [64]. Taken together, these data suggest a link between BPA, β -cell function, and insulin resistance in T2DM [65].

### 5.3. Cardiovascular Toxicity of Bisphenol A

The exposure to BPA may lead to atherosclerosis (ATH) and myocardial infarction and induce arrhythmias [66]. In a zebrafish model, the primary targets for BPA cardiac toxicity were postulated to be located in heart atrioventricular valves [67]. Another study demonstrated that even low concentrations of BPA could affect heart contraction and electrophysiology [68]. Furthermore, myocardial degeneration and arrhythmogenic effects were reported to be linked with BPA exposure, through an ER-mediated mechanism [69]. Recent studies on ATH on mice reported that long-term exposure to BPA can possibly lead to overexpression of several genes involved in cholesterol biosynthesis [70]. Kim et al. demonstrated the atherogenic effect of BPA to be related to an increased level of low-density lipoprotein (LDL) in mice [71].

### 5.4. BPA and Cancer

BPA exposure has been demonstrated to be associated with an increased risk of cancer [72]. More precisely, BPA does not only stimulate neoplastic proliferation in hormone-dependent tumors by acting as a ligand for hormone receptors, but also through direct regulation of oncogenes and/or tumor-suppressor genes. Downregulation of p53 has been observed after exposure to BPA in breast cancer cells, in association with downregulation of the downstream proapoptotic Bcl2-associated X protein (BAX) gene [73]. Moreover, anti-apoptotic effects of BPA are associated to the activation of pro-survival signaling pathways by BPA. For example, Phosphoinositide 3-kinase/Protein kinase B/mechanistic target of the rapamycin (PI3K/Akt/Mtor) pathway is upregulated by BPA, as well as Eukaryotic translation initiation factor 4B (e1F4B) and Eukaryotic translation initiation factor 4E (e1F4E), activators downstream of the mTOR pathway. The activation of this cascade is associated with BPA-mediated resistance to anti-cancer drugs [74]. On the contrary, reduced expression levels of well-known suppressor genes such as Phosphatase and tensin homolog (PTEN), Tuberous sclerosis 1 (TSC1), and Tuberous Sclerosis Complex 2 (TSC2) [74]. BPA also appears to induce mitotic delay of cancer cell lines by perturbing chromosomal congression by disrupting the localization of mitotic regulators (including polo-like kinase 1 Plk1, Kinesin Family Member 2A Kif2a, and Targeting protein for Xklp2 TPX2), therefore, determining multipolar spindle formation through centriole overduplication and premature disengagement [75].

BPA exposure has been described to result in disrupted cell cycle and DNA damage by activating Catenin Beta 1 (CTNNB1), which is the initiator of the aberrant constructed CTNNB1-nuclear factor kappa-B1 (NFKB1)-AR-insulin-like growth factor-1 (IGF1)-Twist-related protein 1 (TWIST1) pathway, potentially leading to lymphomagenesis [76].

With regards to different oncological settings, BPA notably interferes with mammary gland morphogenesis, and thus has a potential oncogenic role in breast cancer development [77]. However, BPA exposure is also related to the development of oral cancer [78], with local high BPA concentrations in the oral cavity being possibly associated with the use of composite resin [79]. BPA may be linked to the pathogenesis of oral and oropharyngeal cancers [80] due to BPA-mediated direct activation of ERs present in the oral mucosa, in the salivary glands [81,82], and in oral cancer cells. BPA agonistic action on ER triggers cell proliferation, invasion, and migration through G-protein related intracellular signaling [83]. Prolonged exposure of BPA was also proven to modify mucosal architecture inducing preneoplastic changes in murine oral cavity [84]. BPA pro-oncogenic role was clarified through in vitro demonstration of BPA-induced expression of metalloproteinases and growth factors, enhancing cell proliferation and angiogenesis, while decreasing apoptosis [85,86,87,88].

### 5.5. Bisphenol A and Infertility

BPA seems to be involved in male infertility by interfering with spermatogenesis through the inhibition of signaling pathways linked to proliferation and stimulation of apoptosis. In particular, apoptosis is induced through the cytochrome C, Bax and caspase-3 and -9 pathways [89]. Furthermore, BPA also results in the accumulation of reactive oxygen species (ROS) in sperm cells, causing damage to the endoplasmic reticulum and mitochondria [90]. At the same time, oxidative stress enhances sperm cell apoptosis through the downregulation of c Bcl-2 expression [91]. Furthermore, BPA interferes with male fertility by decreasing the concentration of free testosterone and, in general, affecting global hormone balance by inhibiting the activity of specific enzymes involved in the production of sex hormones, such as StAR, CYP450scc, CYP45017α, 3β-HSD, and 17β-HSD [92].

BPA determines molecular alterations that also cause female infertility by reprogramming of imprinted genes during postnatal development of oocytes. Based on the literature, BPA exposure seems to have an impact on two maternally-imprinted genes (Igf2r, encoding for insulin-like growth factor 2 receptor and Peg3—Paternally Expressed 3). Insulin-like growth factor II receptor (Igf2r) and paternally expressed 3 (Peg3) are imprinted postnatally from postnatal days (PND) 5 to 25 in the mouse, which is consistent with oocyte and follicle development. Previous studies suggested that there was a close relation between oocyte growth and the establishment of Igf2r and Peg3 imprinting by decreasing their methylation [93]. This BPA-induced hypomethylation is due to ER-mediated DNA Methyltransferases (Dnmts) expression [94]. BPA also interferes with follicular development [95]. Moreover, BPA induces increased expression of BAX in oocytes and concomitantly decreases the expression of LIM Homeobox 8 (Lhx8), folliculogenesis-specific basic helix–loop–helix (Figlα), Spermatogenesis- and Oogenesis-Specific Basic Helix-Loop-Helix-Containing Protein 2 (Sohlh2), and newborn ovary homeobox protein (Nobox) [96].

Moreover, BPA interferes with the meiotic cell cycle by altering the centrosomes and altering the formation of the meiotic spindle during meiosis-I and II [97].

BPA can also target the placenta resulting in a reduced capacity for invasion of trophoblastic cells through alterations in the expression of Epithelial cadherin (E-cadherin), DNMT1, Tissue Inhibitor of Metalloproteinase 1 (TIMP-1) and 2 (TIMP-2), and matrix metalloproteinase-2 (MMP-2) and matrix metalloproteinase-9 (MMP-9). Anti-proliferative and pro-apoptotic effects on the trophoblast seem to be linked to decreased c-myc and increased p53 mRNA expression [98,99].

### 5.6. BPA and Polycystic Ovary Syndrome (PCOS)

BPA has been demonstrated to contribute to the etiology of a number of endocrine disorders, such as male and female infertility, precocious puberty, hormone-dependent tumors (including breast and prostate cancer), and a number of other metabolic disorders [100]. Among these, polycystic ovary syndrome (PCOS) plays a key role. PCOS is the most prevalent endocrinopathy among women of childbearing age and one of the possible factors that trigger such endocrine condition is BPA. It has been demonstrated that women with PCOS have an elevated pulse generator activity of the GnRH, which results in a constant increase of LH, which in turn impairs follicular development and increases ovarian androgen production. Several studies revealed that BPA serum concentrations in PCOS patients were higher compared to healthy controls, which could be possibly linked to gonadotropin impaired secretion. Additionally, BPA has also been proven to directly stimulate androgen synthesis in the ovarian theca-interstitial cells [101].

### 5.7. BPA and the Gut

Recent studies have reported that BPA alters the intestinal epithelium. In more detail, BPA increases intestinal permeability by reducing tight junctions through the downregulation of ZO-1, occludin, claudin-1 and claudin-4 gene expression. Moreover, BPA induces intestinal oxidative stress and reduces antioxidants by decreasing the expression of SOD, GPx, CAT, and T-AOC. Lastly, BPA has been described to activate the gut innate immune system, as demonstrated by the expression of TLR2, TLR4, MyD88, and NF-κB, as well as by increased levels of pro-inflammatory cytokines, such as IL-1β, IL-6, IL-8, and TNF-α [102,103].

### 5.8. BPA and Thyroid Dysfunction

It is well known that BPA has an estrogenic action and is as such considered a representative endocrine disruptor. Several works highlight that BPA can disrupt the thyroid-hormone axis and its action. In fact, in a paper published by Moriyama et al. BPA was described to alter thyroid hormone action. In detail, the authors reported that the similarities between the chemical structures of BPA and triiodothyronine (T_3_) may lead to a reaction with the thyroid receptors, evidencing that BPA can reduce T_3_ binding by suppressing the transcriptional activity of this hormone by recruiting nuclear receptor co-repressors (N-CoRs) [104]. Furthermore, previous in vitro studies suggest that BPA binds to thyroid hormone receptor (TR) alpha and TR-beta, acting like a T_3_ inhibitor [105,106]; The TR-antagonistic action of BPA is probably the principal mechanism through which it interferes with thyroid function [107]. BPA has also been described as possibly being linked to thyroid neoplasms.

Li et al., for example, described a higher incidence of thyroid nodules in Chinese women exposed to BPA [108]. Moreover, a direct relationship between higher urinary BPA levels (due to higher iodine alimentary intake) and papillary thyroid cancer occurrence was demonstrated by recent studies [109].

### 5.9. BPA and Renal Dysfunction/Nephrotoxicity

Kidneys and urinary tracts are also possible targets for BPA action. Renal dysfunction can be related to the urinary excretion of BPA. Based on the literature, several studies evidenced that BPA exposure can induce modifications in renal tissues from a histological point of view [110]. Moreover, renal tissue modifications were also confirmed in animal models: necrosis and atrophy were observed in kidneys from fish exposed to BPA causing [111]. Furthermore, Kobroob et al. hypothesized that BPA may damage kidneys by reducing antioxidant levels, as suggested by high levels of oxidative markers. In more detail, the authors reported that BPA in vivo can affect kidney mitochondrial functions, eventually leading to organ damage. A significant reduction in kidney size after 16 weeks of BPA exposure was also observed [112]. Moreover, previous works highlighted that BPA levels may be used as predictors for chronic kidney disease in patients affected by primary hypertension [113].

## 6. BPA and MSCs

MSCs are a peculiar type of multipotent cells present in mesenchymal tissues [114,115]. MSCs were initially discovered in the bone marrow (BM-MSCs), but afterwards other sources of MSC were identified in other tissues and organs including, among others, lungs, muscles, adipose tissue, placenta, umbilical cord, dermis, and dental tissue.

MSCs are characterized by the expression of specific surface molecules (such as CD90, STRO-1, CD105, CD73), adherence to plastic in culture and the capability of differentiating into chondrocytes, adipocytes, and osteocytes [116,117].

**Figure 3:**
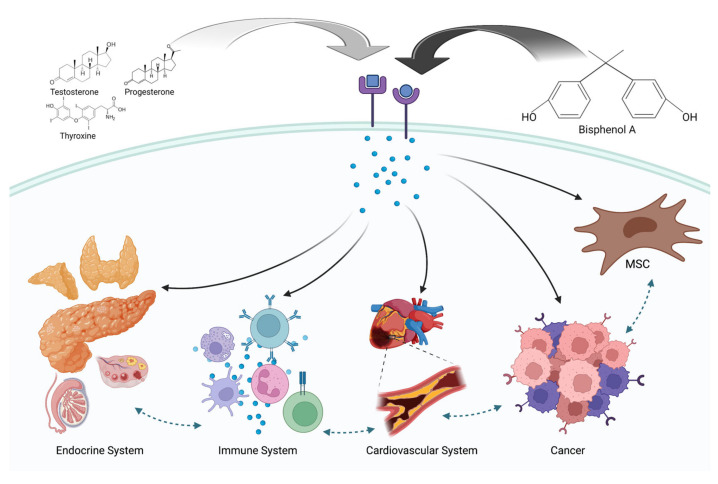
Schematic representation of the main targets for BPA in the human body, including MSCs (mesenchymal stem cells). Created with BioRender.com.

MSCs have recently been discovered to represent potential target cells for BPA (see Figure 3). A recent study evaluated cell viability, apoptosis, DNA damage, and the differentiation potential of adipose tissue derived MSCs (ADSCs) exposed to non-cytotoxic doses of BPA and TCDD [102]. The authors concluded that even low doses of those EDCs were able to reduce cell viability and induce DNA damage, Moreover, adipogenic differentiation significantly varied according to BPA concentration. Other studies confirmed adipogenesis and lipid production to increase in the presence of low doses of BPA (0.1–1 μM), whereas significantly decreasing in the presence of higher levels of BPA [118]. However, contrasting data came from other observations on the synergistic action of Vitamin D and BPA. In fact, an Iranian research group found concentrations of around 10 nM to boost the adipogenic process, which were otherwise inhibited at a concentration of 0.1 nM [119].

Despite the lack of agreement on standardized concentrations for defining BPA levels as “pro-adipogenic”, most of the current literature points at BPA-induced changes as possible key players in driving adipogenesis in MSCs and, therefore, as a possible therapeutic target for the treatment of obesity [120,121,122,123].

Chamorro-García and co-authors hypothesized BPA-mediated differentiation of MSCs to occur through peroxisome proliferator-activated receptor gamma-independent mechanism [124].

Intrauterine exposure to BPA was confirmed to possibly induce obesity in the newborn in animal models, confirming the importance of BPA-mediated action on MSCs in vivo [120]. Other studies highlighted the importance of prenatal BPA-induced epigenetic changes in MSCs possibly driving the adipogenic process [125,126].

Other groups focused on BPA-induced alterations in MSC functions. They observed the activation of myelogenesis-related pathways as a possible effect of BM-MSCs exposed to BPA on the differentiation of hematopoietic stem cells [127].

Recently, research has also been focusing on MSCs as a possible therapeutic tool for the treatment of BPA-induced tissue damage. With this aim, the beneficial effects of MSCs in association with resveratrol were demonstrated in an experimental animal model of BPA-induced uterine damage [128].

Further studies also indicated cytotoxicity and apoptosis to be induced by BPA exposure in rat and human MSCs, possibly affecting their therapeutic capacity [129].

A possible explanation could reside in BPA-mediated superoxide anion overload and reduced β-catenin signaling in MSCs [130].

## 7. Conclusions

BPA is one of the most biologically relevant EDCs and its involvement in different diseases is demonstrated by the association of BPA exposure with thyroid and/or gonadal dysfunction and cancer. However, BPA-mediated effects seem to be implicated in the pathogenesis of a large variety of other disorders, not necessarily directly related to its endocrine disrupting action. These include, among others, immune impairment, obesity, diabetes, cardiovascular diseases, and kidney failure. As for its interaction with molecular targets, BPA acts mainly as a receptor ligand which, as explained above, determines its effect as an endocrine disruption and its involvement with the immune system, diabetes mellitus, myocardial degeneration, and cancer; however, BPA also interferes with other molecular pathways. A special mention has to be granted for BPA-induced adipogenesis in MSCs. In fact, altered MSC function and differentiation associated with BPA exposure could at least partially explain some of the BPA-mediated effects on human health, such as its role in obesity.

However, future studies aimed at better characterizing the clinical significance of environmental concentrations of BPA on MSC function and human health are urgently needed.

## Data Availability

Data are available to the corresponding author upon request.
